# Littoral cell angioma, a rare cause of long standing anaemia: a case report

**DOI:** 10.1186/1757-1626-2-9115

**Published:** 2009-11-30

**Authors:** Danai Chourmouzi, Elsa Psoma, Antonios Drevelegas

**Affiliations:** 1 Department of Diagnostic Radiology, Interbalcan Medical Center, Thessaloniki, Greece; 2 Department of Diagnostic Radiology, Ahepa University Hospital, Thessaloniki Greece

## Abstract

Littoral cell angioma is a rare primary splenic tumor that is difficult to differentiate preoperatively from other benign and malignant splenic lesions. We report a case of littoral cell angioma of the spleen in a 51-year-old woman that presented with long standing anaemia.

## Background

Littoral cell angioma is a tumor of vascular proliferation unique to the spleen. It originates from the cells lining the venous sinuses of the normal spleen, which, under yet unexplained stimuli, proliferate to form the characteristic lesions seen grossly. It has been postulated that these cells react to as yet unknown antigenic stimuli with proliferation and increasing phagocytic activity as a result of their unusual elongated cytoplasmic surface and basement membrane discontinuity. There is a strong association between this neoplasm and a group of immunologic or oncologic entities, including Crohn disease and adenocarcinoma of the colon and pancreas.

This neoplasm occurs most often in middle-aged men and women and has equal sex distribution. From the clinical point of view the most frequent sign is splenomegaly associated with hypersplenism leading to thrombocytopenia and anemia.

We report a new case of littoral cell angioma of the spleen in a 51-year-old woman that presented with long standing anaemia. The imaging findings and the differential diagnosis are discussed.

## Case presentation

A 51-year-old Greek woman was admitted to our hospital with abdominal pain, weakness and fatigue. She described intermittent episodes of left upper quadrant pain with no history of nausea, vomiting, or changes in bowel habits. She had a 5-year history of iron deficiency anaemia that had been treated with oral administration of ferrous iron salts. Beside that, her past medical history was unremarkable except nephrolithiasis.

**Figure 1:**
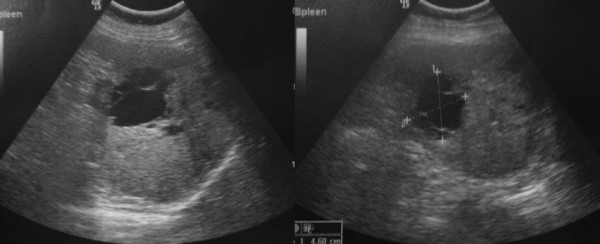
**Ultrasound of the spleen demonstrates enlarged spleen with well-defined hypoechoic, and hyperechoic lesions**.

**Figure 2:**
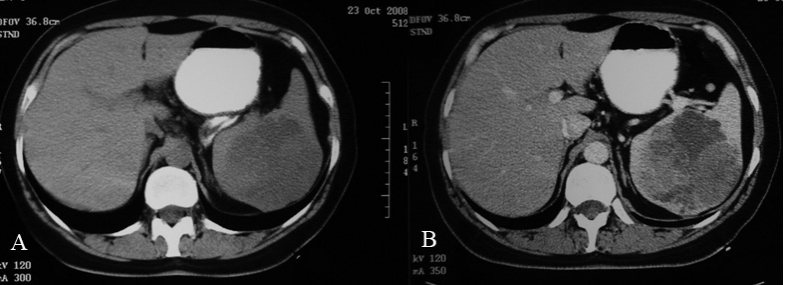
**CT unenhanced (a) and contrast enhanced during the early portal venous phase (b) images show an inhomogeneous hypodense well defined mass with some internal enhancement in the spleen**.

Heavy menstrual bleeding was considered the underlying source of anaemia. However the last two years she was in climacteric period. 12 months had been past after her final menses. On physical examination splenomegaly was noted. The spleen was palpated bimanually with the patient in a supine position 4 cm below the costal margin. Routine laboratory tests showed orthochromic normocytic anemia (haemoglobin 10 g/dl, Ht 31%, MCV 85 fl, MCHC 31 g/dl), with normal levels of WBC (7500/mm), platelets (180000/mm), iron and ferritin. Red cell morphology and white cell differential count, platelet number and morphology on peripheral smear was normal. Colonoscopy and gastroscopy were normal. Ultrasound (US) revealed a large mass with hypoechoic, and hyperechoic component located at the inferior aspect of the spleen (Figure 1). The splenic lesion was further evaluated with CT. An inhomogeneous hypodense well defined mass with some internal enhancement was demonstrated on the contrast enhanced CT scan (Figure 2).

Our presumptive preoperative diagnosis was lymphoma or hemangioma.

Although biopsy of the spleen is a useful procedure in evaluating infectious and neoplastic splenic masses there was concern about potential hemorrhagic complications of the procedure. So the spleen was surgically removed. The patient had an uneventful post-operative recovery.

Pathologic evaluation identified a non-encapsulated but well-circumscribed mass. The lesion consisted of anastomosing vascular channels, lined by histiocytes, with papillary projections and cyst-like spaces. The tumour lining cells were positive for CD31/CD68 markers, and negative for CD34. The findings were compatible with Littoral cell angioma.

Anaemia in this patient with splenomegaly is probably due more to an increase in the splenic plasma pool than to an increase in circulating plasma volume. Six months after surgery, the patient remains asymptomatic without any additional therapy.

## Conclusion

Littoral cell angiomas (LCAs) are rare splenic vascular neoplasms that arise from the cells lining the red pulp sinuses. The definitive diagnosis is made on histology and confirmed with immunohistochemistry. On the cut surface of the spleen, multiple focal nodules of similar size corresponding to sponge like vascular spaces are usually present. Nodules range in colour from red to black, an appearance that reflects the presence of blood products of variable age. These nodules are typically well delineated from surrounding splenic tissue but do not have a surrounding capsule. Tiny cystic spaces associated with the nodular lesions, and corresponding to the vascular spaces seen histologically, may be seen as well. The tumor is characterized by a mixture of papillary and cystic areas lined by neoplastic cells deriving from normal splenic lining-littoral cells. The neoplastic LCA cells express both endothelial and histiocytic antigens associated with CD8 negativity, compared with the normal endothelium of the venous sinuses of the spleen red pulp that only expresses endothelial antigens and CD8 positivity. Therefore, the typical and characteristic immunohistochemical pattern of the LCA is as follows: CD31, CD68, CD163, CD21, FVIII antigen positive; CD34, CD8 negative [1].

The clinical course is benign and in most cases asymptomatic. Signs of hypersplenism with anaemia and thrombocytopenia have been reported. Abdominal pain, pyrexia of unknown origin has been reported. LCA may present as an incidental finding.

One third of cases are associated with tumors of visceral organs, including colorectal, renal, hepatocellular, lung and pancreatic adenocarcinomas, malignant lymphoma, myelodysplastic syndrome, or aplastic anaemia. Close clinical follow-up of patients with LCA of the spleen is recommended [2].

An extensive list of possibilities such as multiple hemangiomas, lymphoma, metastatic disease, and disseminated infections caused by fungi, mycobacteria, *Pneumocystis carinii *and sarcoidosis, should be considered in the differential diagnosis of multinodular splenomegaly [3].

The sonographic appearance of littoral cell angioma is variable and includes reports of mottled echotexture without discrete lesions, as well as findings of isoechoic, hypoechoic, and hyperechoic lesions [4]. On abdominal CT scans obtained with or without contrast material, littoral cell angioma typically manifests as multiple hypoattenuating lesions. Given the vascular nature of these neoplasms, tend to enhance either homogeneously or inhomogeneously [5,6].

On MR images, the nodular lesions of littoral cell angioma typically appear markedly hypointense with both T1- and T2-weighted pulse sequences, a finding that reflects the presence of hemosiderin in the lesions due to the hematophagocytic capacity of the neoplastic cells. However, significant siderosis is seen in less than 50% of LCA cases and the rest of the lesions tend to be hyperintense on the T2 weighted images [7,8].

However, the radiologic features of LCA are rarely diagnostic since many other splenic neoplasms such as hamartomas, haemangiomas, lymphomas, metastatic disease and infectious processes exhibit similar imaging characteristics.

Symptomatic LCA are often relieved by splenectomy, and given the association of LCA with other malignancies and reported cases of metastasizing LCA, splenectomy is both diagnostic and therapeutic. While there have been reports of medical therapy with glucocorticoids and angioembolization of splenic haemangiomas, splenectomy is still considered the gold standard for treatment of vascular splenic tumours.

## Consent

Written informed consent was obtained from the patient for publication of this case report and accompanying images. A copy of the written consent is available for review by the Editor-in-Chief of this journal.

## Competing interests

The authors declare that they have no competing interests.

## Authors' contributions

DC was a major contributor in writing the manuscript. EP and AD performed analysis and interpretation of data. All authors read and approved the final manuscript.
